# Association of Microvasculature and Macular Sensitivity in Idiopathic Macular Epiretinal Membrane: Using OCT Angiography and Microperimetry

**DOI:** 10.3389/fmed.2021.655013

**Published:** 2021-11-16

**Authors:** Jingyang Feng, Xiaotong Yang, Mengqiao Xu, Yuwei Wang, Xiang Shi, Yumeng Zhang, Peirong Huang

**Affiliations:** ^1^Department of Ophthalmology, Shanghai General Hospital (Shanghai First People's Hospital), Shanghai Jiao Tong University School of Medicine, Shanghai, China; ^2^National Clinical Research Center for Eye Diseases, Shanghai, China; ^3^Shanghai Key Laboratory of Fundus Disease, Shanghai, China; ^4^Shanghai Engineering Center for Visual Science and Photomedicine, Shanghai, China; ^5^Department of Ophthalmology, Affiliated Hospital of Nantong University, Nantong, China

**Keywords:** microperimetry, epiretinal membrane, vessel density, prognostic factors, OCT angiography

## Abstract

**Purpose:** To investigate the correlation between retinal capillary structure and macular function in patients with idiopathic epiretinal membrane (iERM) by using optical coherence tomography angiography (OCTA) and microperimetry.

**Methods:** This retrospective and observational study included 30 idiopathic ERM eyes of 30 consecutive patients. OCTA was performed to evaluate macular microvasculature including the superficial capillary plexus, deep capillary plexus, and foveal avascular zone. Best corrected visual acuity (BCVA) and microperimetry were measured at baseline and 3 months after surgery. Associations between macular microvasculature and visual function were assessed.

**Results:** Visual function including BCVA and macular sensitivity improved significantly at 3 months post-operatively (*p* < 0.001). At baseline, BCVA was positively correlated with foveal or parafoveal sensitivities and negatively correlated with central foveal thickness (*p* < 0.05). Pre-operative foveal sensitivity was significantly correlated with the vessel density of foveal or parafoveal superficial capillary plexus (*p* < 0.05). A multiple regression model revealed that pre-operative vessel density of foveal deep capillary plexus was an independent positive prognostic factor for post-operative BCVA (*B* = −0.020 ± 0.006, *p* = 0.006) and macular sensitivity (*B* = 0.200 ± 0.081, *p* = 0.027).

**Conclusion:** Integrated evaluation of iERM by using OCTA and microperimetry shows an association between microvasculature and macular sensitivity. Pre-operative vessel density of foveal deep capillary plexus assessed by OCTA may be a potentially valuable prognostic factor for iERM surgery.

## Introduction

Idiopathic epiretinal membrane (iERM) is characterized by a semi-translucent fibrocellular proliferative membrane at the vitreoretinal interface (1). It occurs mostly in patients over 50 years old with an incidence ranging from 2.2 to 28.9% (2). The proliferation of various cell types such as glial cells, hyalocytes, fibroblasts, and the alterations of vitreous status contributes to the membrane formation (3, 4). An incomplete posterior vitreous detachment (PVD) may provide appropriate conditions for proliferation in the area between the vitreous and the retina (5). Additionally, the dysregulation of vitreous microRNAs (miRNAs) (miR-19b, miR-24, and miR-142-3p) has been reported to be correlated with the fibrosis in eyes affected by ERM (6). The anteroposterior and tangential traction caused by ERM on the inner retina and macular vasculature may induce functional and circulatory disturbance in foveal and/or perifoveal areas, which eventually lead to visual acuity impairment and metamorphopsia (7).

Optical coherence tomography (OCT) has been widely applied in the evaluation of different ocular diseases and in particular to detect any macular or optic disk change in different systemic or macular disorders (8–10), but does not allow detection and visualization of the retinal microvasculature (11). OCT angiography (OCTA) is a more recent development, which makes it possible to visualize the vascular network and blood flow by using an algorithm known as split-spectrum amplitude-decorrelation angiography (12, 13). Compared with fundus fluorescein angiography, OCTA has the advantage of higher resolution, less time-consuming, and non-invasive (14–16). Furthermore, OCTA can visualize blood vessels at various depth-resolved levels including the superficial capillary plexus (SCP), the deep capillary plexus (DCP), and can better delineate the complexities of the vessels at the edge of the foveal avascular zone (FAZ) (17, 18).

In the management of iERM, pars plana vitrectomy (PPV) has become a standard surgical technique. Particularly, recent advances in transconjunctival small-gauge vitrectomy and non-vitrectomizing vitreous technique have provided much potential improvement over conventional 20-gauge surgery such as less surgical-related complications, faster incision healing, better patient comfort, and earlier visual recovery (19, 20). Although best corrected visual acuity (BCVA) is regarded as the main outcome of functional recovery after PPV for ERM, it does not reveal many other functional changes related to macular pathologies. Microperimetry can provide further objective and quantitative information about macular function by evaluating retinal sensitivity in the area of interest (21, 22). It has already shown better efficacy and higher sensitivity compared with BCVA for ERM (23). Moreover, retinal sensitivity has a closer association with reading ability in patients with fundus diseases (24). Only one previous study integrated OCTA with microperimetry to assess iERM eyes (25). However, this study mainly focused on a 1-mm^2^ square foveal region and excluded parafoveal region. Besides, the associations of pre-operative OCTA parameters and post-operative visual function were not investigated.

Thus, in this study, we explored the associations between macular sensitivity and microvascular characteristics in both the foveal and parafoveal regions before and after iERM surgery and to identify the potential prognostic factors indicative of functional recovery.

## Methods

This retrospective and observational study recruited 30 eyes of 30 consecutive patients diagnosed iERM at the Shanghai General Hospital between June 2019 and December 2019 were included. All the patients underwent 23-gauge PPV with ERM/internal limiting membrane (ILM) peeling and fluid-air exchange. Cataract surgery was performed at the time of vitrectomy in all the phakic eyes. All the patients were followed-up for at least 3 months after surgery. The study was compliant with the tenets of the Declaration of Helsinki and was approved by the Ethics Committee of Shanghai General Hospital.

At baseline, all the patients underwent comprehensive ophthalmological examinations including medical history, Snellen BCVA, slit-lamp biomicroscopy, OCTA, and microperimetry. Post-operative visual function including BCVA and microperimetry was also performed at 3 months after surgery.

### Patient Eligibility

Inclusion criteria were as follows: (1) patients with iERM and (2) follow-up at least 3 months. Exclusion criteria were as follows: (1) ERM secondary to other retinal disease, (2) severe cataract or glaucoma, (3) high myopia with a refractive error of more than −6.00 diopters or an axial length longer than 26 mm, (4) retinal vascular disease, (5) a history of previous vitreoretinal surgery, or (6) uncontrolled systemic disease.

### Optical Coherence Tomography Angiography Measurement

Optical coherence tomography angiography was conducted by using the AngioVue Imaging System (RTVue XR Avanti, Optovue Incorporation, Fremont, California, USA), which operates at an A-scan rate of 70,000 scans/s with an 840 nm wavelength. The split-spectrum amplitude-decorrelation angiography algorithm was utilized to record the vascular signals. Based on the default software (version 2015.100.0.35, Optovue Incorporation, Fremont, California, USA), the SCP and the DCP of OCTA images were segmented automatically. The boundary of the SCP was set from 3 μm below the ILM to 16 μm below the inner plexiform layer (IPL). The boundary of DCP was set from 16 to 69 μm below the IPL. Two masked ophthalmologists (authors YXT and SX) reviewed the accuracy of segmentation with a senior retinal specialist (author FJY) consulted in the event of discrepancy. If the automatically segmentation was not proper, manual correction of the segmentation was performed by embedded software in the AngioVue Imaging System (RTVue XR Avanti, Optovue Incorporation, Fremont, California, USA). Vessel density (VD) was defined as the percentage of an area occupied by vessels and calculated automatically by the software on OCTA. Each OCTA scan covered the areas of 1 mm × 1 mm and 3 mm × 3 mm to detect the vasculature of the SCP and DCP in the fovea and parafovea that correspond to the Early Treatment Diabetic Retinopathy Study (ETDRS) macular fields. The central foveal thickness (CFT) was defined as the mean thickness in the central circular area. The FAZ was defined as the area without capillary network in the macula and was measured automatically. Poor quality images with segmentation errors, motion artifacts, or low signal strength index (<50) were excluded. iERM was defined with OCTA B-scans as a thin hyperreflective band anterior to the ILM with focal areas of macular attachments or globally adherent to the retinal surface (26). A staging system reported by Govetto et al. was used to classify iERM (27).

### Microperimetry Evaluation

Following our previous study described (28), MP-1 microperimetry (NIDEK, NAVIS Software version 3.6.4, Gamagori, Japan) was conducted in a dark room with pupil dilation (1% tropicamide) and with the contralateral eye patched. Macular sensitivity was tested at the central 10° with 40 points arranged in three concentric circles (2, 6, and 10°). The Goldmann III stimuli (10 candela/m^2^) were randomly presented for a duration of 200 ms on a 1.27 candela/m^2^ background. Sensitivity (dB, decibels) was assessed by using a 4-2-1 automated staircase strategy ranging from 0 to 20 dB in 2 dB steps. The mean overall macular sensitivity was calculated as the mean threshold of 40 points within the 10° central field. The mean foveal and perifoveal sensitivities were calculated from points within 2° of the center and points within the region from 2 to 10°, respectively.

### Surgical Procedure

A standard three-port 23-gauge PPV was conducted by the same surgeon by using the Constellation Vision System (Alcon, Fort Worth, Texas, USA). After core vitrectomy, the posterior hyaloid was removed from the retina with the assistance of triamcinolone acetonide (TA) (2.5 mg/ml) and a complete vitrectomy was performed at the peripheral vitreous base. The macular ERM was removed by using intraocular forceps and indocyanine green solution (1.5 mg/ml) was applied to stain the ILM within the arcade. The ILM was peeled around the macula to a region of approximately three optic disk diameters. Abundant fluid-air exchange was slowly conducted by using sterilized air. If air leakage occurred through any of the three incisions, a transscleral suture was applied. All the patients were required to maintain a face-down position for at least 3 days after surgery.

### Statistical Analysis

Continuous parameters were presented as mean and SD. The BCVA was converted to the logarithm of the minimum angle of resolution (logMAR). The significant difference in logMAR BCVA before and after surgery was analyzed by using the Wilcoxon signed-rank test. Pre- and post-operative retinal sensitivities were analyzed by using the paired *t*-test. The Pearson correlation analysis was used to analyze the associations between OCTA parameters and post-operative visual outcomes. The multiple linear regression analysis was performed to assess the influence of variables on post-operative visual function. All the statistical analyses were performed by using the Statistical Package for the Social Sciences (SPSS) software (version 21.0, SPSS Incorporation, Chicago, Illinois, USA). *p* < 0.05 was considered to be statistically significant.

## Results

### General Characteristics

This study reviewed the records of 30 iERM eyes from 30 consecutive patients. Three eyes were excluded due to unstable fixation during microperimetry and two eyes were excluded due to low quality of OCTA images. Data from 25 eyes (stage 2: 4 eyes; stage 3: 17 eyes; and stage 4: 4 eyes) of 25 patients (8 males and 17 females) were, therefore, included in analysis. The mean age of the sample was 67.00 ± 8.18 years (range: 55–82 years). The mean axial length (AL) and intraocular pressure (IOP) were 23.12 ± 0.52 mm and 14.20 ± 2.42 mm Hg, respectively. Four eyes (16.0%) were pseudophakic at baseline. The mean pre-operative logMAR BCVA of included eyes was 0.62 ± 0.23 (Table 1). At 3 months post-operatively, the mean logMAR BCVA had increased to 0.39 ± 0.18 (*t* = 5.986, *p* < 0.001).

**Table 1:** Baseline characteristics of study sample.

**Characteristics**	**Baseline (*n* = 25)**
**Age (years)**	
Mean ± SD	67.00 ± 8.18
Range	55 ~ 82
**Gender**	
Male, *n* (%)	8 (32.0%)
Female, *n* (%)	17 (68.0%)
**Side**	
Right, *n* (%)	14 (56.0%)
Left, *n* (%)	11 (44.0%)
**Duration of symptoms (months)**	
Mean ± SD	6.88 ± 3.27
Range	3 ~ 13
Axial length (mm)	23.12 ± 0.52
Intraocular pressure (mmHg)	14.20 ± 2.42
**Lens status**	
Pseudophakic, *n* (%)	4 (16.0%)
**ERM stage**	
Stage 2, *n* (%)	4 (16.0%)
Stage 3, *n* (%)	17 (68.0%)
Stage 4, *n* (%)	4 (16.0%)
**BCVA (logMAR)**	
Mean ± SD	0.62 ± 0.23

*SD, standard deviation; ERM, epiretinal membrane; BCVA, best corrected visual acuity; logMAR, logarithm of the minimum angle of resolution*.

### Optical Coherence Tomography Angiography and Microperimetry Analysis Outcomes

At baseline, VD of the SCP and the DCP, measured by using OCTA, was 44.54 ± 6.45% and 43.54 ± 6.65%, respectively. The mean VD was 38.38 ± 8.55% for the foveal SCP and 44.98 ± 6.58% for the parafoveal SCP. The mean VD was 35.68 ± 6.53% for foveal DCP and 44.85 ± 7.81% for parafoveal DCP. The mean area of FAZ was 0.08 ± 0.06 mm^2^ in the eyes with iERM. The mean CFT of included eyes was 467.44 ± 87.84 μm (Table 2).

**Table 2:** OCTA and microperimetry parameters in idiopathic ERM eyes.

**Parameters**	**Baseline**	**3 Months**	*P*-value^*^
**OCTA parameters**			
**SCP (%)**			
VD of macular SCP	44.54 ± 6.45		
VD of foveal SCP	38.38 ± 8.55		
VD of parafoveal SCP	44.98 ± 6.58		
**DCP (%)**			
VD of macular DCP	43.54 ± 6.65		
VD of foveal DCP	35.68 ± 6.53		
VD of parafoveal DCP	44.85 ± 7.81		
FAZ area (mm^2^)	0.08 ± 0.06		
CFT (μm)	467.44 ± 87.84		
**Microperimetry parameters**			
**Retinal sensitivity (dB)**			
Macular (within 10**°** of center)	10.16 ± 1.79	11.72 ± 2.14	**0.001**
Foveal (within 2**°** of center)	7.61 ± 2.07	9.16 ± 3.22	**0.006**
Parafoveal (2**°** to 10**°** of center)	10.79 ± 1.89	12.36 ± 2.05	**0.001**

*VD, vessel density; SCP, superficial capillary plexus; DCP, deep capillary plexus; FAZ, foveal avascular zone; CFT, central foveal thickness, dB = decibels*.

* *P-value was based on paired t test, significant difference bolded*.

**Figure 1:**
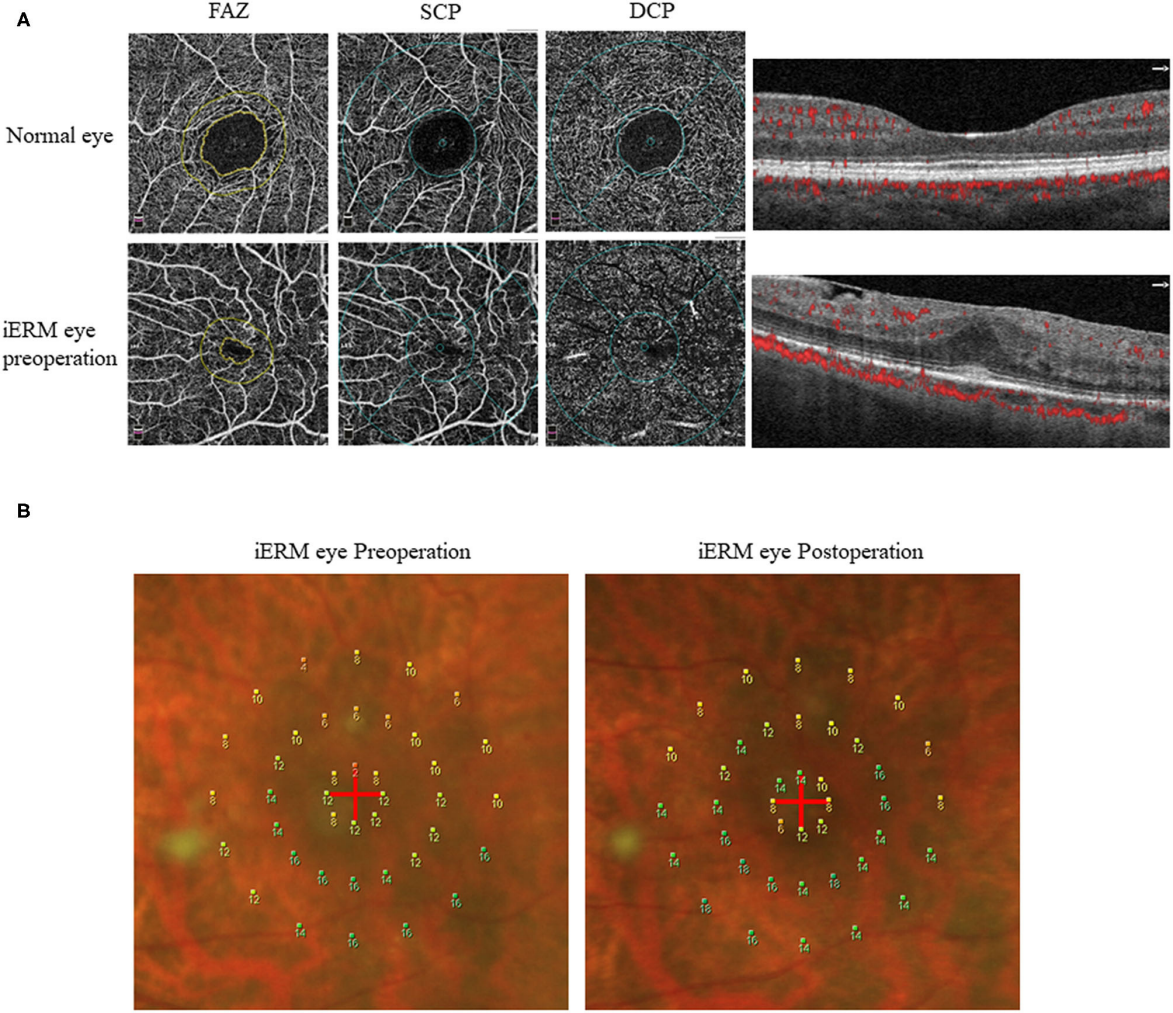
Representative OCTA and microperimetry images. **(A)** OCTA images of a 63-year-old female with iERM (lower row) compared with the normal eye (upper row). **(B)** The microperimetry results from this patient by MP-1 examination before and 3 months after surgery showed improvement.

The mean macular sensitivity (within 10° of center) was 10.16 ± 1.79 dB at baseline and increased to 11.72 ± 2.14 dB at 3 months after surgery (*t* = −3.684, *p* = 0.001). The mean foveal sensitivity (within 2° of center) improved from 7.61 ± 2.07 pre-operatively to 9.16 ± 3.22 dB post-operatively (*t* = −3.006, *p* = 0.006). The mean parafoveal sensitivity (in the region 2° to 10° of center) improved from 10.79 ± 1.89 pre-operatively to 12.36 ± 2.05 dB post-operatively (*t* = −3.585, *p* = 0.001) (Table 2). Representative case is shown in Figure 1.

### Association of OCTA and Microperimetry Parameters in iERM

**Figure 2:**
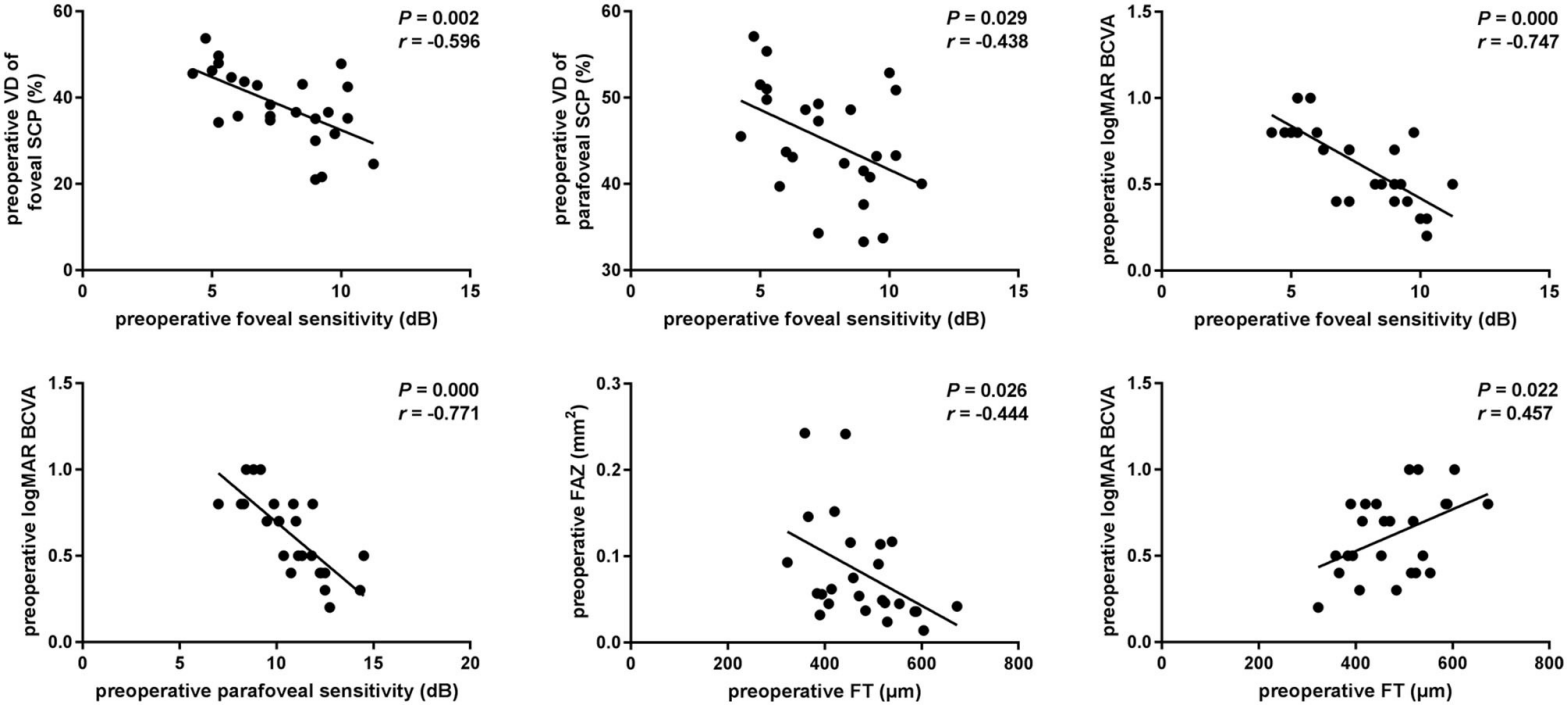
Associations of visual function and OCTA parameters in iERM eyes at baseline. Scatterplots show the statistically significant correlation between preoperative foveal sensitivity and vessel density (VD) of foveal or parafoveal SCP (*r* = −0.596, *P* = 0.002; *r* = −0.438, *P* = 0.029). Preoperative BCVA was significantly correlated with foveal or parafoveal sensitivity, CFT (*r* = −0.747, *P* < 0.001; *r* = −0.771, *P* < 0.001; *r* = 0.457, *P* = 0.022). FAZ area was significantly correlated with CFT (*r* = −0.444, *P* = 0.026).

At baseline, the VD of foveal and the parafoveal SCP was each significantly correlated with foveal sensitivity (*r* = −0.596, *p* = 0.002 and *r* = −0.438, *p* = 0.029, respectively). Statistically significant correlations were also found between pre-operative sensitivity and the baseline logMAR BCVA at the fovea and parafovea (*r* = −0.747, *p* < 0.001 and *r* = −0.771, *p* < 0.001, respectively). Thus, the VD of the SCP reflected the retinal sensitivity in fovea, but not in parafovea. No significant association was found between the SCP and BCVA (*p* > 0.05) indicating that microperimetry may be a more sensitive indicator of changes in the SCP parameters. The mean pre-operative CFT was associated with the mean baseline FAZ and the logMAR BCVA (*r* = −0.444, *p* = 0.026 and *r* = 0.457, *p* = 0.022, respectively) (Figure 2). We did not find any significant correlation between VD of the DCP with foveal or parafoveal sensitivity or the logMAR BCVA at baseline (*p* > 0.05).

### Predictors for Post-operative Visual Functional Recovery

**Figure 3:**
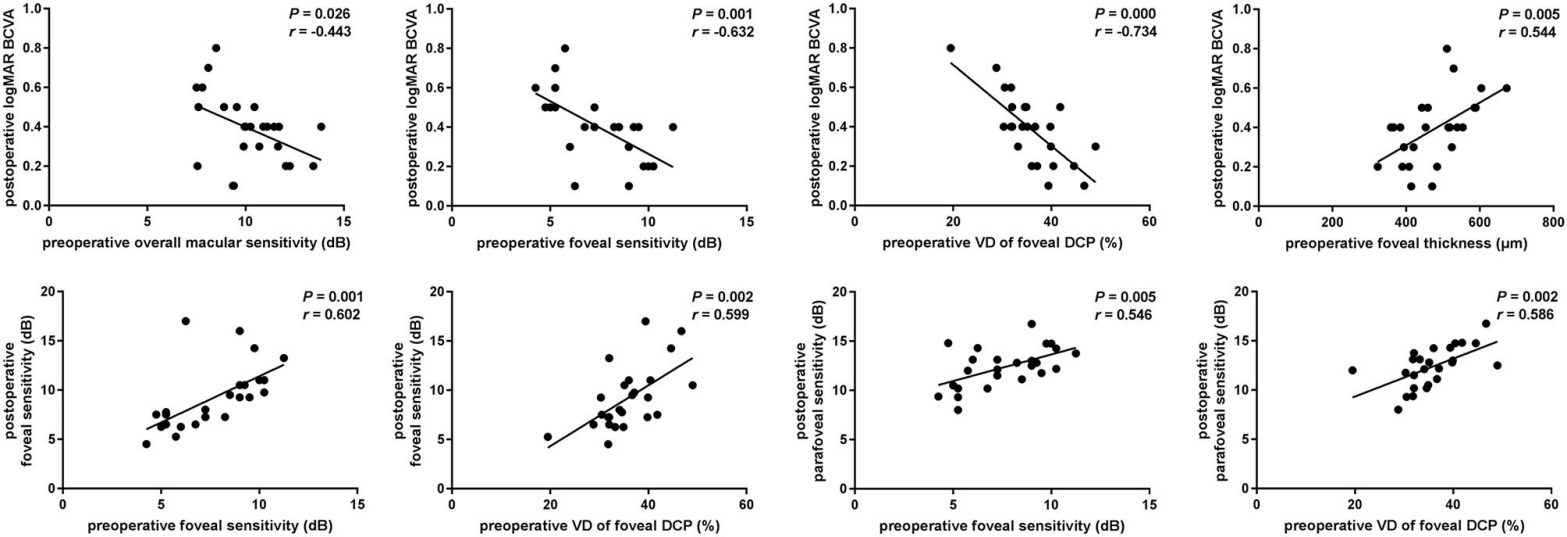
Associations of postoperative visual function and preoperative parameters. Scatterplots show the statistically significant correlation between postoperative logMAR BCVA and preoperative macular or foveal sensitivity, vessel density (VD) of foveal SCP and CFT (*r* = −0.443, *P* = 0.026; *r* = −0.632, *P* = 0.001; *r* = −0.734, *P* < 0.001; *r* = 0.544, *P* = 0.005). Postoperative foveal or parafoveal sensitivity was significantly correlated with preoperative foveal sensitivity and vessel density of foveal DCP (*r* = 0.602, *P* = 0.001; *r* = 0.599, *P* = 0.002; *r* = 0.546, *P* = 0.005; *r* = 0.586, *P* = 0.002).

To explore the predictive factors for visual functional recovery after iERM surgery, correlations between the post-operative logMAR BCVA or retinal sensitivity and pre-operative ocular variables were analyzed (Figure 3). The logMAR BCVA at 3 months after surgery was significantly correlated with pre-operative overall macular or foveal sensitivity (*r* = −0.443, *p* = 0.026 and *r* = −0.632, *p* = 0.001, respectively), baseline VD of the foveal DCP (*r* = −0.734, *p* < 0.001), and the CFT (*r* = 0.544, *p* = 0.005). The foveal sensitivity at 3 months after surgery was significantly correlated with pre-operative foveal sensitivity (*r* = 0.602, *p* = 0.001) and baseline VD of the foveal DCP (*r* = 0.599, *p* = 0.002). The parafoveal sensitivity at 3 months after surgery was correlated with pre-operative foveal sensitivity (*r* = 0.546, *p* = 0.005) and baseline VD of the foveal DCP (*r* = 0.586, *p* = 0.002). There was no correlation between post-operative visual function and pre-operative FAZ (*p* > 0.05).

Based on these findings, the multiple linear regression model, adjusted for age and gender, was applied to screen the prognostic factors for visual functional recovery after iERM surgery. We found that pre-operative VD of the foveal DCP was an independent predictor for the post-operative logMAR BCVA (*B* = −0.020 ± 0.006, *p* = 0.006) and mean macular sensitivity (*B* = 0.200 ± 0.081, *p* = 0.027) at 3 months after surgery (Table 3).

**Table 3:** Multiple linear regression model of post-operative logMAR BCVA or macular sensitivity and baseline ocular parameters.

**Baseline parameters**	**Post-operative logMAR BCVA**				**Post-operative macular sensitivity**			
	***B***	***Se (B)***	*P* ^*^	**95% CI**	***B***	***Se (B)***	*P* ^*^	**95% CI**
Age (years)	−0.005	0.004	0.194	−0.014 to 0.003	0.073	0.051	0.175	−0.037 to 0.182
Gender	0.050	0.083	0.555	−0.128 to 0.229	0.243	1.082	0.826	−2.077 to 2.563
BCVA (logMAR)	0.219	0.218	0.333	−0.249 to 0.687	4.273	2.836	0.154	−1.809 to 10.355
Foveal sensitivity (dB)	0.001	0.023	0.957	−0.048 to 0.051	0.321	0.301	0.304	−0.325 to 0.967
Parafoveal sensitivity (dB)	−0.017	0.028	0.558	−0.078 to 0.044	0.578	0.368	0.138	−0.211 to 1.368
VD of foveal SCP (%)	−0.002	0.004	0.649	−0.010 to 0.006	0.038	0.050	0.465	−0.070 to 0.146
VD of parafoveal SCP (%)	0.006	0.005	0.270	−0.005 to 0.017	−0.076	0.065	0.266	−0.216 to 0.064
VD of foveal DCP (%)	−0.020	0.006	**0.006**	−0.034 to −0.007	0.200	0.081	**0.027**	0.026 to 0.374
VD of parafoveal DCP (%)	0.004	0.004	0.352	−0.004 to 0.012	−0.009	0.049	0.864	−0.115 to 0.097
CFT (μm)	0.000	0.001	0.789	−0.001 to 0.001	−0.005	0.007	0.998	−0.015 to 0.015

*Se (B), standard error of B coefficient; BCVA, best corrected visual acuity; logMAR, logarithm of the minimum angle of resolution; VD, vessel density; SCP, superficial capillary plexus; DCP, deep capillary plexus; CFT, central foveal thickness; dB, decibels*.

* *P-value was based on multiple linear regression model, significant difference bolded*.

*Adjusted R^2^ for post-operative logMAR BCVA = 0.627, for post-operative macular sensitivity = 0.577*.

## Discussion

We have provided a novel report on the association between microvasculature and macular sensitivity both the foveally and parafoveally before and after surgery for iERM. Our findings included: (1) The VD of foveal or the parafoveal SCP was negatively correlated with foveal sensitivity in iERM eyes, but not with BCVA and (2) Pre-operative VD of the foveal DCP was an independent positive prognostic factor for post-operative visual function including BCVA and macular sensitivity.

The relationship between morphology and function is an interesting and meaningful topic. However, few studies focused on morpho-functional evaluation by combining the OCTA and microperimetry. The correlation between VD and post-operative retinal function has been reported in full-thickness macular holes (29). For iERM, only one study investigated the post-operative VD of the SCP, the DCP, and retinal sensitivity (25). However, in contrast with this study, no OCTA-based pre-operative predictors were identified for ERM surgeries. A possible explanation for this discrepancy is the shape and range of macular region measured in the two studies. This study used a circular shape including the foveal and parafoveal regions, while the previous study (25) used a square shape excluding the parafoveal region. According to former study, 1° apart from fovea is approximately equal to 300 μm in human eyes with normal AL (30). Using this metric, the diameters of 2, 6, and 10° in the microperimetry are around 600, 1,800, and 3,000 μm. In the OCTA, the foveal circle has a diameter of 1,000 μm and the parafoveal area is an annulus with inner diameter of 1,000 μm and an outer diameter of 3,000 μm. These dimensions allow 2° retinal sensitivity in the microperimetry to be fully covered by foveal VD in the OCTA and the 6 and 10° dimensions are fully covered by the parafoveal vessel density. Thus, the foveal and parafoveal VD have a well-geographical correspondence with 2, 6, and 10° retinal sensitivity, respectively.

Visual prognostic factors of ERM surgery are varied and inconsistent in previous study with duration of symptoms, central bouquet (CB) alteration staging, and existence of pseudoholes having been reported as possible predictive factors (31, 32). In long-term prognosis, BCVA at baseline, but not classification of CB, appeared to be a strong predictor for BCVA improvement following surgery (26). The CFT has been identified as a significant predictor in some study (31), but not others (33, 34). In this study, we found that the CFT was associated with post-operative visual function, but the multiple linear regression analysis showed that it was not a significant predictor for the post-operative logMAR BCVA or macular sensitivity.

The inner retinal changes play a relatively important role in the pathology of ERM as the tangential traction imposed by the membrane occurs at the inner interface of the retina (35). Consistent with this, inner retinal deformation has been found to be the sole factor associated with significant long-term improvement in vision (36). These findings suggest that OCTA, which reflects inner retinal vessel flow (including the SCP and the DCP), could help in identify new prognostic factors for ERM surgical outcomes. This newly developed technique has the advantages of safety and speed of en face or transverse images of retinal vessel vasculature without dye injection.

Based on our observations, while the VD of foveal or the parafoveal SCP was negatively corrected with visual function at baseline, pre-operative VD of the foveal DCP but not the SCP was an independent positive prognostic factor for post-operative BCVA and macular sensitivity at 3 months after surgery. There are several possible explanations for this phenomenon: (1) Prior to surgery, greater distortion by the membrane leads to more of the vascular network being pulled into the central macular region. Outer retinal damage is derived from inner retinal change through tangential inward traction at the fovea and retrograde transneuronal degeneration (37); (2) After surgical removal of the ERM and ILM by microincision vitrectomy with gas tamponade, the traction force was alleviated and a flattening force was imposed and the vessels in the foveal returning to their original location. This process is reversible in most cases and could explain why the pre-operative SCP is not a prognostic factor for iERM surgery; and (3) The DCP vessels are close to the outer nuclear layer and photoreceptors, acting as the most important source of blood delivery to the outer retina. Due to edema, ischemia, and distortion in the pathological process, the flow of the DCP decreased, exacerbating damage to the outer retina and causing irreversible photoreceptor dysfunction. Lin et al. found that the tractional forces of ERM not only distorted the SCP, but also affected the DCP (38). Other studies also observed the defect of cone outer segment tips and abnormal microfolds of photoreceptor layer in eyes with ERM (36, 39). These findings indicate that tractional forces initiated from the inner retina could reach as far as the photoreceptor layer. It seems feasible that changes in VD of the DCP are secondary to tractional forces. The decreased VD of the DCP indicated a reduced blood supply to the outer retina, which further exacerbated the pathological progress of iERM.

The pre-operative FAZ area is reportedly correlated with post-operative FAZ area in the patients with ERM (40), but the relationship between the pre-operative FAZ and the post-operative logMAR BCVA was not explored in this study. Our multiple linear regression model showed that the FAZ area is not a significant predictor of the post-operative logMAR BCVA or macular sensitivity.

This study was limited by its relatively small sample. In addition, some patients underwent cataract surgery at the same time as ERM surgery, potentially introducing differences in outcomes within the group. However, previous study has shown that parameters are similar in patients with or without cataract surgery at each follow-up time point (1 month to 2 years) after iERM surgery (25).

In conclusion, this study investigated the morpho-functional relationships in iERM eyes and found correlations between retinal sensitivity and vessel density. Higher pre-operative VD of the foveal DCP is a prognostic factor for better visual function recovery after iERM surgery. Thus, it is useful to measure VD of the DCP by OCTA while evaluating surgical management and prognosis of iERM eyes.

## Data Availability Statement

The original contributions presented in the study are included in the article/supplementary material, further inquiries can be directed to the corresponding author/s.

## Ethics Statement

The studies involving human participants were reviewed and approved by Ethics Committee of Shanghai General Hospital. The patients/participants provided their written informed consent to participate in this study. Written informed consent was obtained from the individual(s) for the publication of any potentially identifiable images or data included in this article.

## Author Contributions

JF, PH, and XS contributed to the study design and protocol. XY conducted the examinations. MX, JF, and PH prepared and reviewed the manuscript. YW and YZ collected and did the statistical analysis of the data. All authors contributed to the article and approved the submitted version.

## Funding

This study was supported by the Shanghai Public Health System's Talent Development Plan (GWV-10.2-YQ09) and the Bethune-Langmu Eye Research Fund (BJ-LM2018001J).

## Conflict of Interest

The authors declare that the research was conducted in the absence of any commercial or financial relationships that could be construed as a potential conflict of interest.

## Publisher's Note

All claims expressed in this article are solely those of the authors and do not necessarily represent those of their affiliated organizations, or those of the publisher, the editors and the reviewers. Any product that may be evaluated in this article, or claim that may be made by its manufacturer, is not guaranteed or endorsed by the publisher.
